# Supplementary figures and images for: Tracking urban human activity from mobile phone calling patterns
Source: PLoS Comput Biol. 2017 Nov 21;13(11):e1005824. doi: 10.1371/journal.pcbi.1005824 (PMC5697809; doi:10.1371/journal.pcbi.1005824)

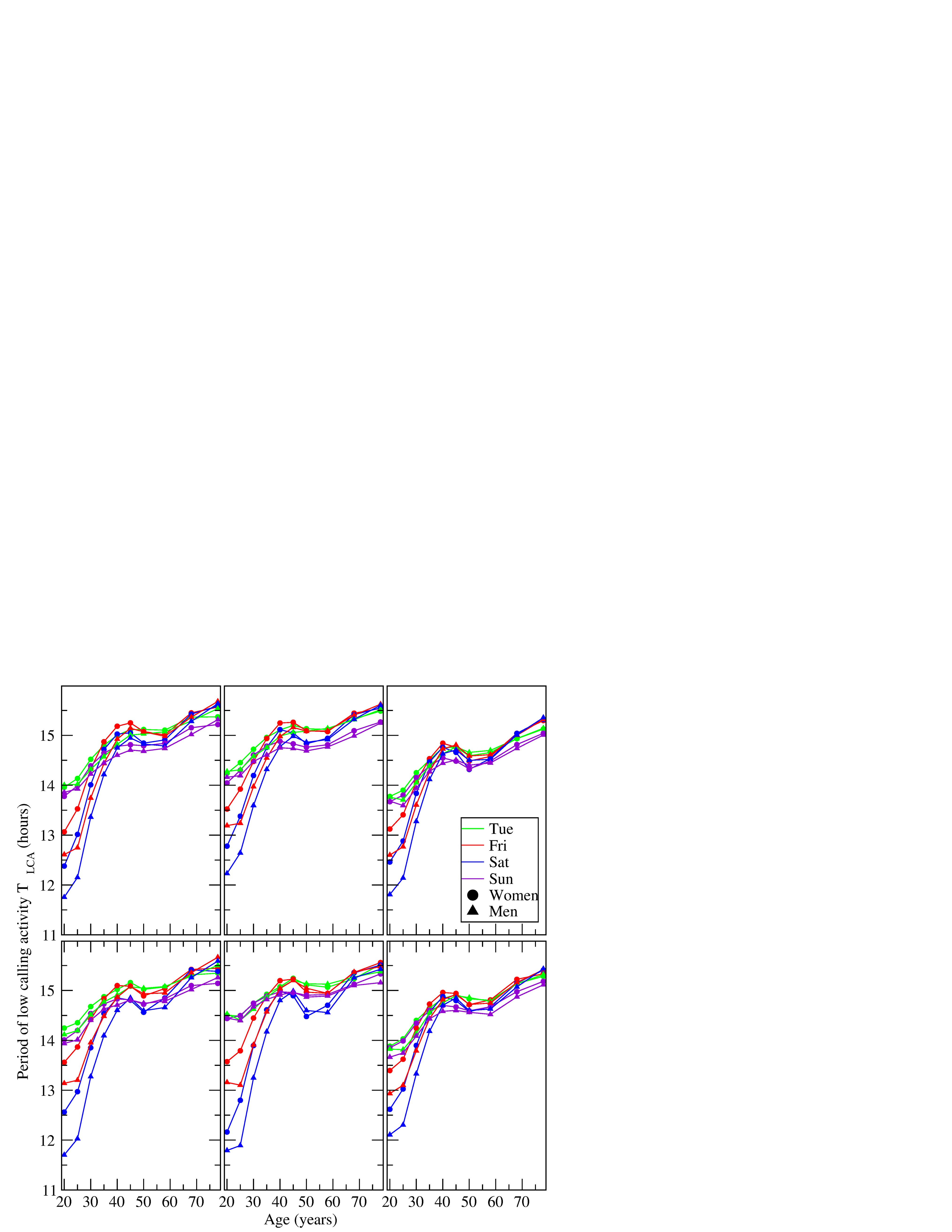

Supplement: S1 Fig — The TLCA is calculated as the elapsed time between the mean time of the last call and of the first call, as a function of the age and gender of different cohorts, for the six most populated city in the dataset in 2007. For each age cohort, TLCA is calculated for females (circles) and males (triangles) separately. TLCA is different for different days of the week, and the corresponding plots are shown for (green) Tuesdays, (red) Fridays, (blue) Saturdays, and (violet) Sundays. Mondays to Thursdays have similar values, therefore only the data for Tuesdays is shown. (TIF) [file pcbi.1005824.s001.tif]

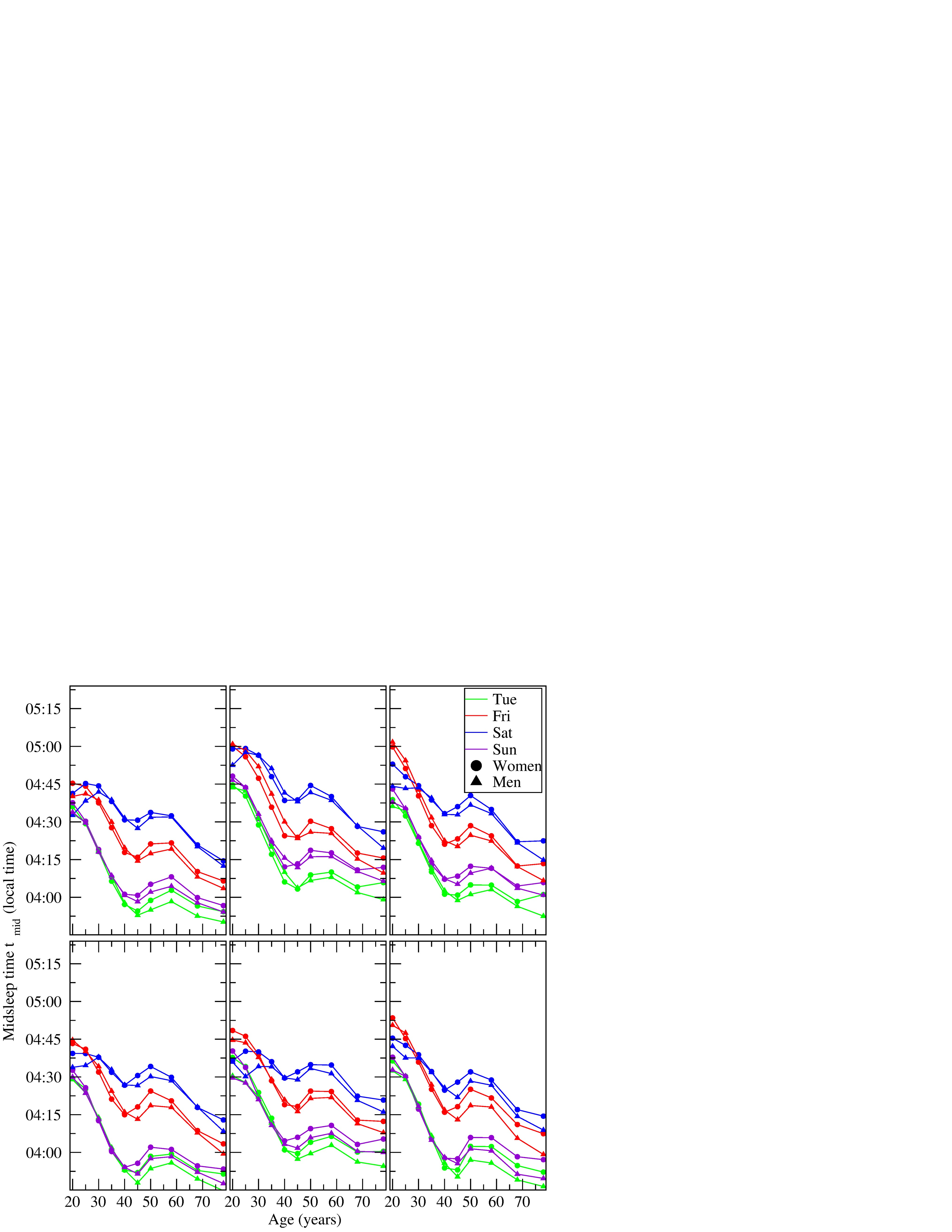

Supplement: S2 Fig — tmid is calculated as the time at middle of the interval between the mean time of the last call and of the first call, as a function of the age and gender of different cohorts, for six of the seven most populated cities in the dataset in 2007. For each age cohort, tmid is calculated for females (circles) and males (triangles) separately. tmid is different for different days of the week, and the corresponding plots are shown for (green) Tuesdays, (red) Fridays, (blue) Saturdays, and (violet) Sundays. Mondays to Thursdays have similar values, therefore only the data for Tuesdays is shown. During some Fridays nights, the calling activity extended until very late in the night, and the distribution of the morning calling activity on the next day presents a small peak around 4:00 a.m. If present, we include this peak in the analysis when calculating the time of the first call, due to its small amplitude and width compared with the main part of the distribution for the time of the first call. This is also true for the results shown in Fig 5 in the main text. (TIF) [file pcbi.1005824.s002.tif]

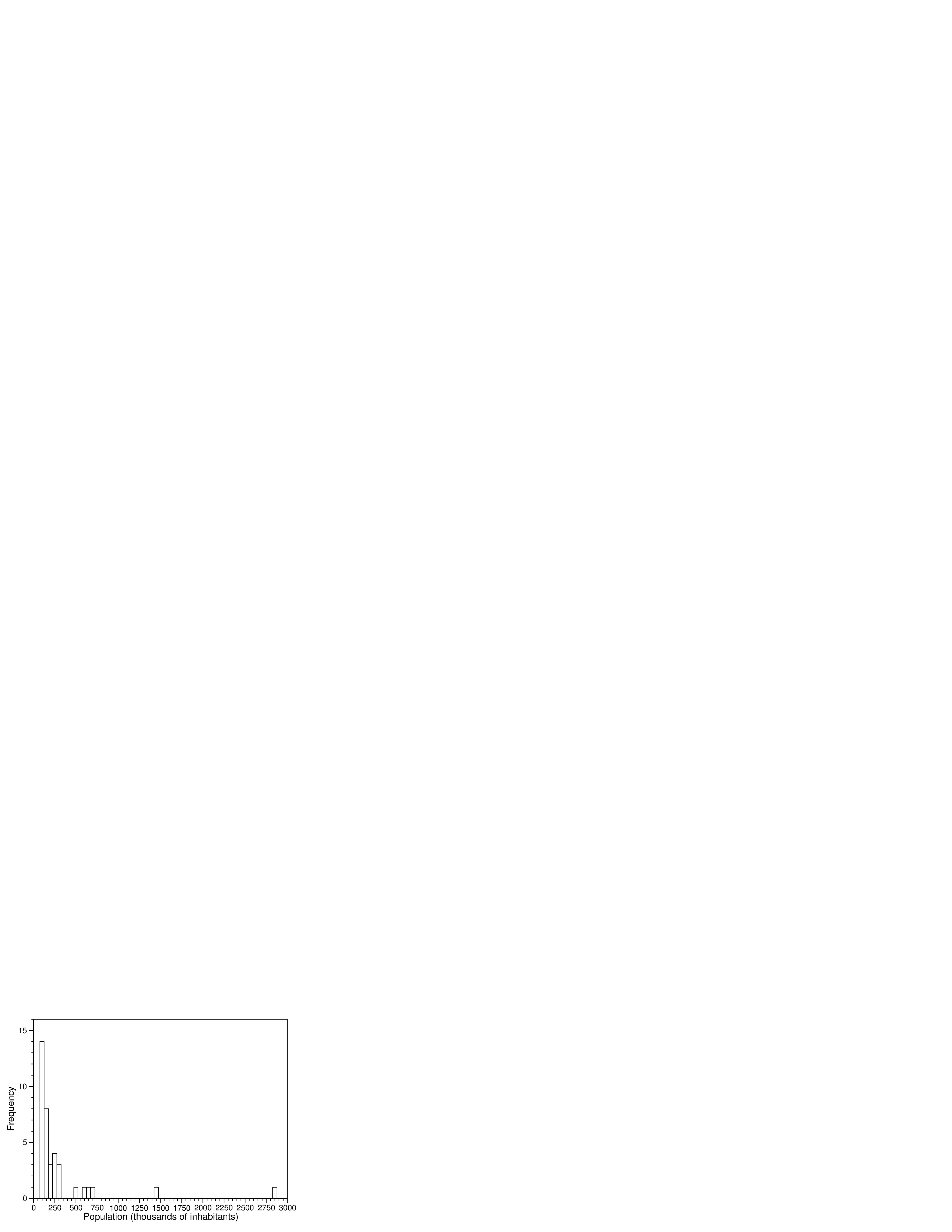

Supplement: S3 Fig — The values are rounded to multiples of 50,000 to keep the identity of each city unknown. (TIF) [file pcbi.1005824.s003.tif]
